# Harmonizing the COVID-19 cacophony: People need guidance

**DOI:** 10.1017/ice.2020.105

**Published:** 2020-04-03

**Authors:** Taha Bin Arif, Aiman Ali

**Affiliations:** MBBS, Dow Medical College, Dow University of Health Sciences, Karachi, Pakistan

*To the Editor*—The COVID-19 pandemic caused by a novel coronavirus (SARS-CoV-2) has sounded danger alarms across the globe. According to a report by the World Health Organization (WHO), >292,142 confirmed cases had been identified worldwide as of March 21, 2020.^1^ Although some differences in the real-world mortality rate and the mortality rate were reported in a few published clinical series, a total of 1,772 deaths with an average mortality rate of 2.5% was reported by the Chinese Center for Disease Control and Prevention on February 16, 2020.^2^ With the rise in global anxiety, the WHO declared COVID-19 the sixth international public health emergency on January 30, 2020. This pandemic could have devastating outcomes across the world if the viral spread is not controlled by public health efforts.

The origin of coronavirus, a single-stranded enveloped RNA virus, dates back in 1960s. The first coronaviruses discovered caused infectious bronchitis in chickens. Two other viruses obtained from nasal secretions of human patients with common colds were named human coronavirus 229E and human coronavirus OC43. Subsequently, other members of this family have been identified, including SARS-CoV in 2003, HCoV NL63 in 2004, HKU1 in 2005, MERS-CoV in 2012, and SARS-CoV-2 (formerly named 2019-nCoV) in 2019.^3^ Most of these virus species cause severe respiratory infections in humans.

Overall, the clinical features of COVID-19, caused by SARS-CoV-2, correspond to common influenza. The clinical manifestations of COVID-19 pneumonia, as demonstrated by some studies, have varied among cases. Fever is the predominant symptom followed by cough, dyspnea, myalgia, headache, and diarrhea.^1^ An effective cure of COVID-19 by antiviral therapy and vaccination is currently being developed. However, considering previous management plans for MERS and SARS infections, the WHO has recommended preventive measures to inhibit the transmission of acute respiratory infections. These interventions include (1) avoiding close contact with people suffering from common cold; (2) frequent cleaning of hands with running water or alcohol sanitizers; (3) avoiding excessive touching of eyes, nose, and mouth; and (4) avoiding unprotected contact with animals. In addition, people with acute respiratory infections should practice respiratory hygiene regularly, which includes maintaining a distance of 1 m from healthy people to avoid spread of airborne droplets, covering coughs and sneezes with clothing or disposable tissues, and frequent hand washing.^1^

Since the emergence of SARS-CoV-2, various international bodies have debated the use of N95 respirators and surgical masks. In their review on the use of masks and respirators for prevention of influenza transmission, Reza et al^4^ reported that effectiveness of masks is likely linked to their early, regular, and correct use; most studies have reported a reduced risk of upper respiratory infections with masks and respirators. Similarly, Jefferson et al^5^ reviewed the clinical effectiveness N95 respirators (95%) and masks (68%) in reducing the spread of respiratory viruses. In a debate organized by the Singapore Infection Control Association, Dr Lin proposed the adequacy of surgical masks for prevention of MERS-CoV transmission in addition to personal and environmental hygiene. Moreover, during SARS outbreak, N95 respirators were perceived to be difficult to tolerate by healthcare workers due to their association with impaired mental performance and headache. According to Professor Seto, the WHO has recommended the use of surgical masks for people living with infected patients and N95 respirators for aerosol-generating procedures. In 2009, the US CDC recommended the use of N95 respirators in all circumstances; however, with evolution of coronavirus pandemics, the guidelines were revised in 2010 and N95 respirators were recommended only for aerosol-generating procedures.^6^ With increasing global apprehension concerning the spread of coronaviruses, people are rushing to Amazon and local pharmacies to buy surgical masks and N95 respirators, resulting in a shortage. Different social media celebrities are also promoting a dangerous myth by unnecessarily using these masks. Considering that N95 respirators filter out 95% of small particles, medical professionals need to wear these masks during close contact with patients. However, they are of no use for general public because these heavy particles can be transmitted through surfaces and, to a limited extent, through air. In a recent press conferences, Alex Azar issued a statement that the United States needs ~300 million N95 respirators for healthcare workers due to the rapid increase in prevalence of this infection.^7^ The paucity of masks and respirators has led to significant price escalation. Some businesses in United States are completely sold out of masks despite evidence that healthy people do not need to wear them.^8^

According to the Centers for Disease Control and Prevention, frequent hand washing with running water and soap for at least 20 seconds, using hand sanitizer, avoiding large gatherings and crowds, and disinfecting fomites may primarily protect others from the virus. However, the eminent global shortage and high prices of masks cannot be overstated. The respective authorities and governments need to develop and implement the proper guidelines and preventative strategies and make sure that the general public is adhering to the proposed protocols. Governments should also provide accurate information and should clarify misinformation spread by social media to help the public face this pandemic.

## References

[1] Novel coronavirus (COVID-19) situation report-62. World Health Organization website. https://www.who.int/docs/default-source/coronaviruse/situation-reports/20200322-sitrep-62-covid-19.pdf?sfvrsn=f7764c46_2. Published March 21, 2020. Accessed April 3, 2020.

[2] Ji Y , Ma Z , Peppelenbosch MP , Pan Q . Potential association between COVID-19 mortality and healthcare resource availability. Lancet Global Health 2020;8(4):e480. 32109372 10.1016/S2214-109X(20)30068-1 PMC7128131

[3] Geller C , Varbanov M , Duval RE . Human coronaviruses: insights into environmental resistance and its influence on the development of new antiseptic strategies. Viruses 2012;4:3044–3068. 23202515 10.3390/v4113044 PMC3509683

[4] bin-Reza F , Lopez Chavarrias V , Nicoll A , Chamberland ME . The use of masks and respirators to prevent transmission of influenza: a systematic review of the scientific evidence. Influenza Other Respir Viruses 2012;6:257–267. 22188875 10.1111/j.1750-2659.2011.00307.x PMC5779801

[5] Jefferson T , Del Mar C , Dooley L , et al. Physical interventions to interrupt or reduce the spread of respiratory viruses: systematic review. BMJ 2009;339:b3675. 19773323 10.1136/bmj.b3675 PMC2749164

[6] Chung JS , Ling ML , Seto WH , Ang BS , Tambyah PA . Debate on MERS-CoV respiratory precautions: surgical mask or N95 respirators? Singapore Med J 2014;55:294. 25017402 10.11622/smedj.2014076 PMC4294054

[7] Celebrities like Gwyneth Paltrow and Kate Hudson are wearing masks to protect against the coronavirus, but they’re promoting a dangerous myth. Business Insider website. https://www.businessinsider.com/coronavirus-celebrities-wearing-masks-are-promoting-a-myth-2020-2. Published February 27, 2020. Accessed April 3, 2020.

[8] Mask prices ramp up on Amazon amid coronavirus outbreak. Daily Mail website. https://www.dailymail.co.uk/news/article-8056879/Mask-prices-ramp-Amazon-sell-stores-amid-coronavirus-outbreak.html. Published February 28, 2020. Accessed April 3, 2020.

[9] Coronavirus disease 2019 (COVID-19)—prevention and treatment. Centers for Disease Control and Prevention. https://www.cdc.gov/coronavirus/2019-ncov/prepare/prevention.html. Updated April 2, 2020. Accessed April 3, 2020.

